# Identifying factors associated with mental health status following climate-related disasters: a nationwide longitudinal panel study in Korea

**DOI:** 10.4178/epih.e2025014

**Published:** 2025-03-27

**Authors:** Eunjin Oh, Jaelim Cho, Changsoo Kim, Hyungryul Lim, Kyoung-Nam Kim

**Affiliations:** 1 Department of Preventive Medicine and Public Health, Ajou University School of Medicine, Suwon, Korea; 2 Department of Preventive Medicine, Yonsei University College of Medicine, Seoul, Korea; 3 Institute for Environmental Research, Yonsei University College of Medicine, Seoul, Korea; 4 Institute of Human Complexity and Systems Science, Yonsei University, Incheon, Korea

**Keywords:** Depression, Anxiety, Post-traumatic stress disorders, Cyclonic storms

## Abstract

**OBJECTIVES:**

Despite the increasing frequency and intensity of climate-related disasters, identifying factors associated with mental health status remains challenging. This study aimed to determine the factors linked to symptoms of depression, anxiety, and post-traumatic stress disorder (PTSD) following heavy rainfall and typhoons.

**METHODS:**

National data on climate-related disaster victims (n=825 for heavy rainfall and n=1,220 for typhoon) from a longitudinal panel in Korea (“Long-term Survey on the Change of Life of Disaster Victims”) and data from individuals unaffected by disasters (n=893) were used. Generalized linear mixed models were employed to evaluate the factors associated with mental health status following climate-related disasters.

**RESULTS:**

Greater disaster severity (e.g., experiencing casualties or asset loss) was associated with higher scores for depression (Patient Health Questionnaire-9), anxiety (Generalized Anxiety Disorder-7), and PTSD (Impact Event Scale-Revised). The association between casualty experience and anxiety score was more pronounced among individuals over 65 years (β [log-transformed score], 1.39; standard error [SE], 0.26; p<0.001), female respondents (β, 1.20; SE, 0.20; p<0.001), those with a low education level (β, 1.18; SE, 0.25; p<0.001), and those with a low income (β, 1.45; SE, 0.26; p<0.001) compared to their counterparts.

**CONCLUSIONS:**

These findings may help guide targeted interventions and shape public health policies and disaster management strategies that prioritize mental health support for the most at-risk populations, ultimately increasing community resilience to climate-related challenges.

## GRAPHICAL ABSTRACT

[Fig f4-epih-47-e2025014]

## Key Message

We identified factors associated with depression, anxiety, and PTSD following climate-related disasters, highlighting that greater disaster severity correlates with worse mental health outcomes. Vulnerable groups, such as older adults, women, those with lower education and income, are at higher risk. The findings suggest the need for targeted mental health interventions and policies to support these populations in future disaster responses.

## INTRODUCTION

Due to climate change, the frequency and intensity of climate-related disasters have been continually increasing. Between 2010 and 2020, climate-related disasters resulted in over 410,000 casualties and impacted approximately 1.7 billion people [1]. Heavy rainfall and typhoons are the most frequent climate-related disasters, with recent reports indicating an upward trend in their occurrence across various regions [2]. A continuous rise in temperature is expected to intensify heavy rainfall events [3] and strengthen typhoons [4]; thus, these disasters represent a substantial threat to human health.

Climate-related disasters have been reported to negatively impact mental well-being [5,6]. People exposed to climate-related disasters may experience mental health problems such as depression within the first year and exhibit chronic mental illness even 2 years after the disaster [7]. Heavy rainfall and typhoons are reported to increase the risk of depression, anxiety, and post-traumatic stress disorder (PTSD) [8-11].

Victims of heavy rainfall and typhoons who experience physical injury or the loss of family members, property, or social support may display a higher risk of mental health problems compared to those who do not face these issues [12-17]. This may be due to psychological stress and feelings of hopelessness, which lead to deteriorated resilience and a reduced capacity for mental health recovery [8]. Although identifying factors associated with mental health status following climate-related disasters (e.g., experiencing casualties or asset loss) is crucial for planning public health policies and prioritizing healthcare resources to minimize impacts, research on these factors remains largely insufficient.

The Korea is particularly vulnerable to climate-related disasters because of its geographical location, with 3 of its 4 borders facing the sea and a climate characterized by concentrated summer rainfall. According to the International Disaster Database, from 1900 to 2014, heavy rainfall and typhoons were the most damaging climate-related disasters in Korea [18]. Therefore, using data from a nationwide panel survey on climate-related disasters and a control group of unaffected individuals in Korea, we explored the factors associated with mental health status following exposure to heavy rainfall and typhoons.

## MATERIALS AND METHODS

### Study participants

This study analyzed data from the “Long-term Survey on the Change of Life of Disaster Victims” (CLDV) conducted by the National Disaster Management Research Institute of Korea. The survey covered Korean victims of disasters (2012-2017) such as heavy rainfall, typhoons, earthquakes, and fires. Participants were systematically selected according to sex, age, and residence, with quotas for each disaster type [19]. Up to 4 surveys were conducted from 2016 to 2019, starting with a baseline survey administered in 2016. Participants were enrolled in the baseline survey due to disasters that occurred that year or up to 4 years prior. The sampling frame was divided by region (Supplementary Material 1), and weights were calculated to ensure regional balance. Data were collected through face-to-face interviews by trained interviewers.

In addition to the CLDV data, individuals not affected by disasters were recruited and surveyed cross-sectionally from 2017 to 2019 using a protocol resembling that of the CLDV, thus representing a control group. This effort was also conducted by the National Disaster Management Research Institute of Korea. In 2017, the control group was sampled from the same cities (the “*si-gun-gu*” level), while excluding the municipal regions (the “*eup-myeon-dong*” level) that were primarily affected by natural disasters. In 2018, individuals who experienced the 2017 Pohang earthquake but did not sustain any damage were included. In 2019, disaster-naive individuals were recruited nationwide.

**Figure 1.**
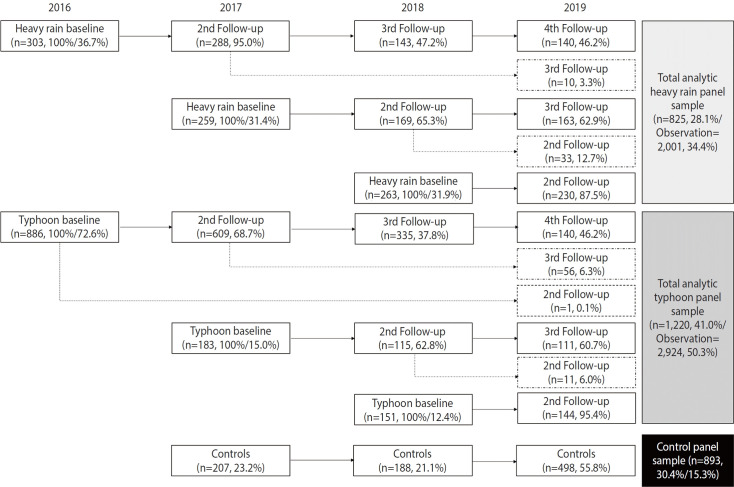
Flowchart of participant recruitment and longitudinal follow-up surveys for victims of heavy rainfall and typhoons, as well as the control group (2016-2019).

Participants (from both the CLDV and the control group) who had previously been diagnosed with psychiatric disorders, including depressive disorders, anxiety disorders, or PTSD, were excluded from the study. Disaster victims were followed up over 4 years, whereas the control participants were surveyed only once. The final sample consisted of 2,938 individuals: 825 (28.1%) heavy rainfall victims, 1,220 (41.0%) typhoon victims, and 893 (30.4%) control respondents (Figure 1).

### Disaster severity

Six variables from the questionnaire were used to describe the severity of climate-related disasters experienced by victims: (1) whether the respondent or nearby individuals were casualties (yes or no); (2) self-reported disaster-induced losses (moderate or less, high, or very high); (3) whether the respondent had to relocate, be separated from their family, or reside in temporary housing because of the disaster (yes or no); (4) whether household income decreased because of the disaster (yes or no); (5) whether household assets decreased because of the disaster (yes or no); and (6) whether household debt increased because of the disaster (yes or no).

### Mental health status

Based on previous research [20], the severity of depressive, anxiety, and PTSD symptoms (continuous variables) was selected as the outcome of interest. Depressive symptoms were assessed using the Patient Health Questionnaire-9 (PHQ-9), which comprises 9 questions about depressive symptoms experienced over the past 2 weeks, with responses on a 4-point scale [21]. Anxiety symptoms were evaluated using the Generalized Anxiety Disorder-7 (GAD-7), which includes 7 questions about anxiety symptoms experienced over the past 2 weeks, also rated on a 4-point scale [22]. PTSD symptoms were assessed using the Impact Event Scale-Revised (IES-R), which consists of 22 items related to emotions and thoughts about a traumatic event experienced over the past week, with responses rated on a 5-point scale [23].

We also defined depression, anxiety, and PTSD as binary variables based on the suggested thresholds of the epidemiological tools (PHQ-9, GAD-7, and IES-R) and considered these as secondary outcomes. For the PHQ-9, individuals with a total score of 9 or higher out of a maximum of 27 were classified as having depression. For the GAD-7, individuals scoring 5 or higher were defined as having anxiety. For the IES-R, individuals scoring 24 or higher were classified as having PTSD.

In this study, while the PHQ-9 and GAD-7 were administered to both disaster victims and the control group, the IES-R was administered only to disaster victims. Accordingly, analyses using the IES-R were conducted among disaster victims alone, with disaster severity serving as the exposure variable.

### Covariates

We selected the following variables as potential confounding factors or predictors of the outcomes and adjusted for them in the analyses: age (year), sex (male or female), region of Korea (North, Midwest, Southwest, Mideast, or Southeast), marital status (single, married, or separated/divorced/widowed), education level (middle school or below, high school, or college or above), and monthly household income (<2, 2-<4, or ≥4 million Korean won [KRW]). Information on covariates was obtained through a structured questionnaire.

### Statistical analysis

Descriptive analyses were performed to examine the socio-demographic characteristics of the climate-related disaster victims and the control group. The mean scores of the PHQ-9, GAD-7, and IES-R, along with the proportions of individuals with depression, anxiety, and PTSD, were presented for the years following the disaster.

To assess the associations of disaster severity with depressive, anxiety, and PTSD symptoms, we constructed generalized linear mixed models (GLMMs) with a negative binomial distribution to account for the right-skewed distribution of the outcomes. We incorporated random intercepts for individuals and time since the ments and the passage of time. Baseline age, sex, region, marital status, education level, and monthly household income were included as fixed effects. Variance inflation factors (VIFs) were calculated to assess collinearity among the fixed-effect covariates. Additionally, we constructed GLMMs with a binomial distribution, using binary variables for depression, anxiety, and PTSD based on the cut-off scores of the PHQ-9, GAD-7, and IES-R, respectively, instead of continuous scores.

To identify vulnerable groups, we stratified the analyses by age (<65 vs. ≥65 years), sex (male vs. female), marital status (single vs. partnered), education level (high school or less vs. higher), and monthly household income (<2 vs. ≥2 million KRW).

In the sensitivity analyses, we first repeated our analyses examining the associations between disaster severity and mental health status, restricting the study population to participants enrolled in the survey within 2 years of disaster occurrence. Second, we excluded 188 controls enrolled in 2018 (Figure 1) who may have been indirectly affected by the 2017 Pohang earthquake from the main analysis. Third, we explored the associations between disaster severity and mental health status by examining the effect heterogeneity between heavy rainfall and typhoons. A 2-tailed paired z-test was used to compare the estimated β coefficients for each disaster.

In the analyses using GLMMs with continuous PHQ-9, GAD-7, and IES-R scores as outcomes, the values of β and standard error (SE) were based on log-transformed scores of the PHQ-9, GAD-7, and IES-R. All analyses were conducted using R version 4.3.1 (R Foundation for Statistical Computing, Vienna, Austria), with the lme4 package used for GLMM analyses.

### Ethics statement

The protocol for this study was approved by the Institutional Review Board of Hanyang University (IRB No. HYU-2023-125). All participants provided written informed consent, and the surveys were conducted in accordance with the principles of the Declaration of Helsinki.

## RESULTS

### General characteristics of study participants

From 2016 to 2019, 63.3% of heavy rainfall victims experienced disasters in 2017, with 38.2% and 35.5% residing in the midwestern and northern regions of Korea, respectively. Meanwhile, 55.0% of typhoon victims were affected in 2012, with 55.6% residing in the southwest region. Furthermore, differences in age, marital status, education level, and monthly household income were observed between the disaster victims and the control group. Specifically, the disaster victims tended to be older, were more likely to be married, and had lower education levels and household incomes (Table 1).

**Figure 2.**
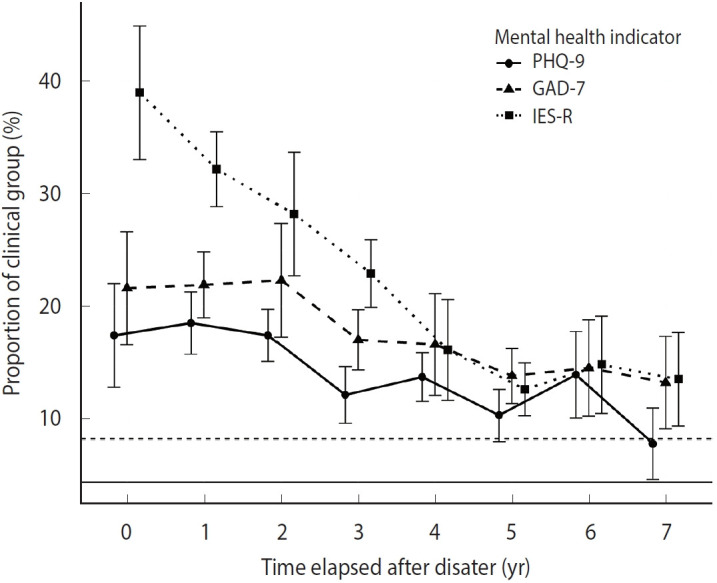
Longitudinal trends in the proportion of depression, anxiety, and post-traumatic stress disorder (PTSD) among climate-related disaster victims tracked for 7 years post-disaster. This figure shows the proportions of climate disaster victims with depression (circles), anxiety (triangles), and PTSD (squares). Depression, anxiety, and PTSD were defined as follows: depression, PHQ-9 score ≥9; anxiety, GAD-7 score ≥5; and PTSD, IES-R score ≥24. Vertical error bars represent standard errors. Horizontal solid and dashed lines show the proportions of the control group with depression and anxiety, respectively. Each wave of surveying targeted disasters that occurred during the survey year or up to 4 years prior. PHQ-9, Patient Health Questionnaire-9; GAD-7, Generalized Anxiety Disorder-7; IES-R, Impact Event Scale-Revised.

The PHQ-9, GAD-7, and IES-R scores, along with the corresponding proportions of individuals with depression, anxiety, and PTSD, declined over time following a disaster. However, even after 5 years or more had elapsed, the proportions of individuals with depression or anxiety remained slightly higher than those in the control group. At 7 years post-disaster, the proportions of individuals with depression and anxiety were 7.8% versus 4.4% and 13.2% versus 8.2% in victims versus controls, respectively (Figure 2, Supplementary Material 2).

### Identification of factors associated with mental health status following climate-related disasters

In the GLMM analyses, which incorporated 5,818 observations from 2,938 individuals, increased disaster severity was associated with higher PHQ-9, GAD-7, and IES-R scores. In all models, the VIF values were below 3 (data not shown). After adjusting for covariates, victims who experienced casualties had higher PHQ-9 scores (β, 0.87; SE, 0.11; p<0.001) and higher GAD-7 scores (β, 1.02; SE, 0.15; p<0.001) than the control group. Similarly, compared to control respondents, victims who experienced post-disaster asset loss also had higher PHQ-9 (β, 0.98; SE, 0.11; p<0.001) and GAD-7 (β, 1.09; SE, 0.15; p<0.001) scores. IES-R scores were higher for victims who experienced casualties (β, 0.51; SE, 0.06; p<0.001) and for those who experienced post-disaster asset loss (β, 0.47; SE, 0.06; p<0.001) compared to victims who did not encounter these issues (Table 2).

In the covariate-adjusted logistic regression models, personally experiencing casualties or being nearby was associated with greater odds of depression (OR, 14.88; 95% CI, 8.08 to 27.38), anxiety (OR, 6.33; 95% CI, 3.99 to 10.03), and PTSD (OR, 3.49; 95% CI, 2.71 to 4.51) compared to the control group. Asset loss was also associated with higher odds of depression (OR, 7.98; 95% CI, 4.78 to 13.32), anxiety (OR, 5.81; 95% CI, 3.80 to 8.89), and PTSD (OR, 2.57; 95% CI, 2.06 to 3.20), consistent with the results of the main analyses obtained using continuous PHQ-9, GAD-7, and IES-R scores (Supplementary Material 3).

**Figure 3.**
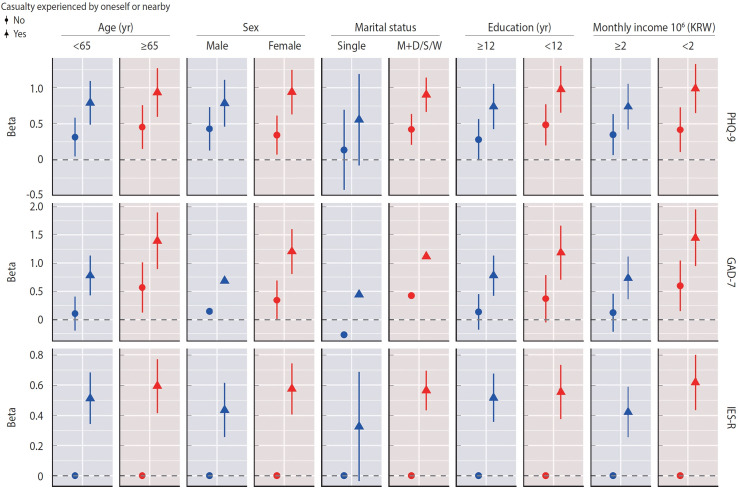
Effect modification by socio-demographic characteristics on the estimates of depression, anxiety, and post-traumatic stress disorder (PTSD) scores among victims with or without casualties, compared to the control group. Estimates were obtained using negative binomial mixed models, adjusting for age, sex, region, marital status, education level, and monthly household income. The analytical models included random effects for individual identification and time since the disaster. Circles represent beta estimates for victims without casualties, triangles denote those with casualties, and vertical lines indicate confidence intervals. Missing vertical lines indicate models failed to estimate standard errors of beta coefficients. GAD-7, Generalized Anxiety Disorder-7; IES-R, Impact of Event Scale-Revised; M+D/S/W, married, divorced, separated, or widowed group; PHQ-9, Patient Health Questionnaire-9; KRW, Korean won.

Stratified analyses by age, sex, marital status, education level, and monthly household income revealed that the association between casualty experience and GAD-7 score was stronger among individuals aged 65 and older (β, 1.39; SE, 0.26; p<0.001), female participants (β, 1.20; SE, 0.20; p<0.001), respondents with a low education level (β, 1.18; SE, 0.25; p<0.001), and individuals with a low household income (β, 1.45; SE, 0.26; p<0.001) compared to their counterparts (Figure 3, Supplementary Material 4).

### Sensitivity analysis

In the sensitivity analysis restricted to participants who enrolled in the survey within 2 years of experiencing a disaster, the results remained robust (Supplementary Material 5). Similarly, even when the 2018 control group—who may have been indirectly affected by the 2017 Pohang earthquake—was excluded, the findings were consistent with those of the main analysis (Supplementary Material 6). Subgroup analysis by disaster type revealed consistent effects on IES-R scores; however, the effect point estimates for heavy rainfall victims were slightly higher than those for typhoon victims, although almost all p-values for heterogeneity did not indicate significance (Supplementary Material 7). This difference may be explained by the fact that most heavy rainfall victims were affected in 2017, while most typhoon victims were impacted earlier, in 2012 (Table 1).

## DISCUSSION

In analyses examining factors related to mental health status following climate-related disasters, we found that higher disaster severity, as indicated by experiencing casualties or asset loss, was associated with greater PHQ-9, GAD-7, and IES-R scores.

Previous research has reported that exposure to climate-related disasters is associated with the development or exacerbation of depression, anxiety, and PTSD [24-27]. According to a panel study conducted in Bangladesh, exposure to heavy rainfall within 12 months prior to the survey was linked to higher PHQ-9 and GAD-7 scores [28]. A United Kingdom study of 819 individuals affected by heavy rainfall found that the prevalence of depression was elevated by 8.48 times (95% CI, 1.04 to 68.97) and PTSD by 7.74 times (95% CI, 2.24 to 26.79) compared to an unaffected group [29].

Previous studies have identified factors that increase vulnerability to mental and physical health issues following exposure to climate-related disasters, such as social isolation and economic instability [30-32]. These findings partially align with those of the present study. Our research extends previous work by conducting more comprehensive analyses, revealing that vulnerability following climate-related disasters was greater among older adults, females, and low-income individuals.

Older adults have been identified as a vulnerable population to climate-related disasters, potentially due to pre-existing health conditions and reduced mobility [33]. Mental health-related emergency visits among older adults have reportedly been elevated immediately after hurricanes (by 32%) and at 3 months, 1 year, 2 years, and 3 years after the event (by 2, 9, 15, and 10%, respectively) [34]. Previous research on flood victims revealed that females were more likely than men to experience PTSD (OR, 3.34; 95% CI, 1.23 to 9.06) and anxiety (OR, 2.90; 95% CI, 1.12 to 7.53) even 17 years after the disaster [35]. For individuals with lower socioeconomic status, disaster-induced economic shocks that exceed their assets can more severely exacerbate mental health issues [36] and heighten distress and concerns about the future following a disaster [37]. Furthermore, higher income has been associated with a lower likelihood of persistent negative mental health outcomes (OR, 0.927; 95% CI, 0.923 to 0.932) [38].

The findings of this study suggest that interventions for heavy rainfall and typhoon disasters might benefit from prioritizing vulnerable groups, such as older adults, females, and low-income individuals. These populations are more likely to experience psychological impacts following climate-related disasters; thus, targeted intervention strategies that address their needs and support mental health recovery could be beneficial. This approach may help allocate resources more effectively for high-risk groups and strengthen recovery pathways.

The biological mechanisms underlying the associations between climate-related disasters and depressive, anxiety, and PTSD symptoms are not fully understood. However, several hypotheses have been proposed. Physical damage, family disruptions, food shortages, and increased conflict caused by disasters may disrupt circadian rhythms and the normal functioning of the hypothalamic-pituitary-adrenal axis [39,40]. In the long term, disasters may induce structural changes in the amygdala and prefrontal cortex through chronic stress and circadian disruptions, leading to allostatic overload and mental health disorders such as depression and anxiety [41]. Additionally, high humidity from heavy rainfall and typhoons can trigger stress or acute stress responses [42,43].

Our study had several limitations. First, because mental health status was assessed through surveys, we were unable to distinguish between clinical and subclinical depression, anxiety, and PTSD. Although we defined these conditions based on the suggested thresholds of the epidemiological tools used, concerns about outcome misclassification persist. Second, disaster severity was evaluated using self-reported questions that may reflect victims’ subjective perceptions of damage rather than the objective severity or extent of disasters. Additionally, because the surveys were not conducted immediately after the disasters, recall bias is possible. We partially addressed this exposure misclassification by leveraging the strength of repeated-measures data, which allowed us to track changes over time. Furthermore, we incorporated various variables to capture multiple aspects of disaster-related damage and observed consistent patterns of association. Additionally, using self-reports to assess disaster severity offers advantages in terms of feasibility, cost-effectiveness, rapid data acquisition, and the ability to reflect individual differences. Future studies should integrate self-reports with objective indicators to obtain a more comprehensive understanding of disaster severity. Third, while the mental health assessment tools used tracked symptoms over only the prior 2 weeks, they are widely used to assess long-term effects [44,45]. Additionally, not all climate-related disasters were covered in this study. Moreover, although the data used in this research are epidemiologically validated, it is important to recognize the limitations of relying on subjective survey data and the regional constraints of the sample.

Nevertheless, to our knowledge, this study is among the first to examine factors associated with mental health status following climate-related disasters using a longitudinal design. By utilizing panel data that followed participants up to 4 times between 2016 and 2019, we were able to identify risk factors and obtain more reliable results that clearly demonstrate exposure-response temporality. In addition, because we used nationwide data that recruited disaster victims based on sex, age, and residence, with quotas for disaster types and individuals unaffected by disasters, the results of this study can be generalized to the broader population of Korea rather than to specific subgroups alone.

This research suggests that greater disaster severity may be associated with poorer mental health outcomes following climate-related disasters. These findings could guide targeted interventions and inform public health policies and disaster management strategies that prioritize mental health support for populations that may be at greater risk (e.g., older adults and individuals with lower education or income levels). Ultimately, such approaches may help increase community resilience to climate-related challenges.

## Figures and Tables

**Figure f4-epih-47-e2025014:**
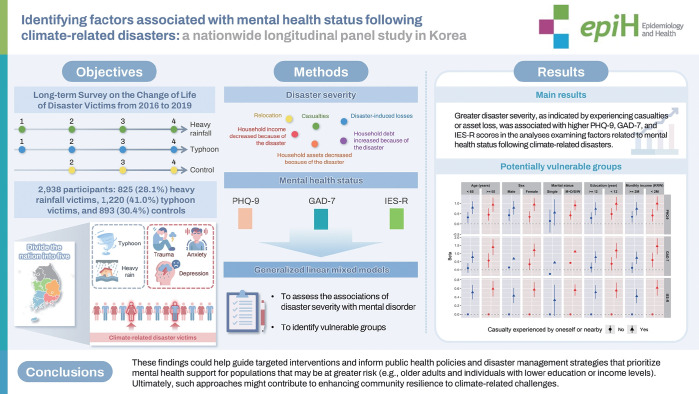


**Table 1.** Socio-demographic characteristics of study participants

Characteristics	Total participants (n=2,938, 100%)	Victims		Control group (n=893, 30.4%)	p-value^1^
		Heavy rain (n=825, 28.1%)	Typhoon (n=1,220, 41.5%)		
Sex					<0.001
Male	1,411 (48.0)	412 (49.9)	623 (51.1)	376 (42.1)	
Female	1,527 (52.0)	413 (50.1)	597 (48.9)	517 (57.9)	
Age, mean±SD (yr)	55.9±17.9	53.4±18.9	60.6±17.7	51.8±15.8	<0.001
Region					<0.001
Capital region (North)	506 (17.2)	293 (35.5)	57 (4.7)	156 (17.5)	
Chungcheong region (Midwest)	523 (17.8)	315 (38.2)	66 (5.4)	142 (15.9)	
Honam region (Southwest)	835 (28.4)	26 (3.2)	678 (55.6)	131 (14.7)	
Gyeongbuk region (Mideast)	470 (16.0)	-	156 (12.8)	314 (35.2)	
Gyeongnam region (Southeast)	604 (20.6)	191 (23.2)	263 (21.6)	150 (16.8)	
Year^2^					<0.001
2012	698 (23.8)	27 (3.3)	671 (55.0)	-	
2013	110 (3.7)	70 (8.5)	40 (3.3)	-	
2014	239 (8.1)	191 (23.2)	48 (3.9)	-	
2015	142 (4.8)	15 (1.8)	127 (10.4)	-	
2016	441 (15.0)	-	334 (27.4)	107 (12.0)	
2017	622 (21.2)	522 (63.3)	-	100 (11.2)	
2018	188 (6.4)	-	-	188 (21.1)	
2019	498 (17.0)	-	-	498 (55.8)	
Marital status					<0.001
Single	532 (18.1)	211 (25.6)	172 (14.1)	149 (16.7)	
Married	1,974 (67.2)	476 (57.7)	862 (70.7)	636 (71.2)	
Separated/divorced/widowed	431 (14.7)	138 (16.7)	185 (15.2)	108 (12.1)	
Education level					<0.001
≤Middle school	1,225 (41.7)	293 (35.5)	691 (56.6)	241 (27.0)	
High school	1,138 (38.7)	363 (44.0)	365 (29.9)	410 (45.9)	
≥College	575 (19.6)	169 (20.5)	164 (13.4)	242 (27.1)	
Average monthly household income (10^6^ KRW)					<0.001
<2	868 (32.8)	290 (35.8)	417 (44.2)	161 (18.0)	
2-<4	1,110 (41.9)	352 (43.5)	369 (39.1)	389 (43.6)	
≥4	669 (25.3)	168 (20.7)	158 (16.7)	343 (38.4)	

Values are presented as number (%). SD, standard deviation; KRW, Korean won.

1 Mean comparisons were performed using a t-test, while frequency comparisons were conducted using chi-square tests.

2 Within the victim group, this refers to the year of disaster occurrence, while in the control group, it denotes the year of survey participation.

**Table 2.** Factors related to poor mental health after exposure to climate-related disasters^1^

Disaster intensity variables	Total (n=2,938)	Surveyed (n=5,818)	PHQ-9 (mean±SD: 1.8±1.6)		p-value	GAD-7 (mean±SD: 1.0±1.3)		p-value	IES-R (mean±SD: 3.7±3.4)		p-value
Control	893 (30.4)	893 (15.3)	1.1±1.2	Reference		0.7±1.0	Reference		-	-	
Casualties experienced by oneself or nearby											<0.001
No	1,697 (57.8)	4,037 (69.4)	1.8±1.5	0.39 (0.10)	<0.001	0.9±1.3	0.31 (0.14)	0.021	4.5±3.1	Reference	
Yes	348 (11.8)	888 (15.3)	2.7±1.8	0.87 (0.11)	<0.001	1.7±1.7	1.02 (0.15)	<0.001	10.0±3.2	0.51 (0.06)	
Self-reported disaster-induced losses											
≤Moderate	565 (21.3)	1,550 (28.0)	1.6±1.6	0.43 (0.11)	<0.001	0.9±1.3	0.42 (0.15)	0.004	4.2±3.2	Reference	
High	671 (25.3)	1,744 (31.6)	1.9±1.6	0.47 (0.11)	<0.001	1.0±1.4	0.39 (0.15)	0.007	5.1±3.1	0.04 (0.06)	0.516
Very high	518 (19.6)	1,340 (24.2)	2.2±1.6	0.59 (0.11)	<0.001	1.1±1.5	0.61 (0.15)	<0.001	6.3±3.1	0.21 (0.06)	0.001
Relocation, separation from family, and residing in temporary housing											<0.001
No	1,734 (59.0)	4,166 (71.6)	1.9±1.5	0.42 (0.11)	<0.001	0.9±1.4	0.36 (0.14)	0.010	4.7±3.1	Reference	
Yes	311 (10.6)	759 (13.0)	2.2±1.8	0.77 (0.12)	<0.001	1.3±1.6	0.79 (0.15)	<0.001	8.6±3.3	0.34 (0.07)	
Household income^2^											<0.001
No change/Increased	1,345 (45.8)	3,535 (60.8)	1.6±1.5	0.39 (0.10)	<0.001	0.8±1.3	0.32 (0.14)	0.022	4.5±3.2	Reference	
Decreased	700 (23.8)	1,390 (23.9)	3.3±1.5	0.92 (0.11)	<0.001	1.5±1.6	0.94 (0.15)	<0.001	7.4±3.1	0.39 (0.05)	
Household assets^2^											<0.001
No change/Increased	1,695 (57.7)	4,043 (69.5)	1.7±1.5	0.41 (0.10)	<0.001	0.8±1.3	0.34 (0.14)	0.014	4.5±3.1	Reference	
Decreased	350 (11.9)	882 (15.2)	3.3±1.7	0.98 (0.11)	<0.001	1.9±1.6	1.09 (0.15)	<0.001	9.6±2.9	0.47 (0.06)	
Household debt^2^											<0.001
No change/Decreased	1,792 (58.9)	4,343 (72.3)	1.9±1.6	0.45 (0.11)	<0.001	0.9±1.4	0.37 (0.14)	0.008	4.9±3.1	Reference	
Increased	314 (10.7)	712 (12.2)	2.4±1.7	0.81 (0.12)	<0.001	1.4±1.5	0.92 (0.16)	<0.001	7.2±3.4	0.35 (0.07)	

Values are presented as number (%), mean±SD or β (standard error). GAD-7, Generalized Anxiety Disorder-7; IES-R, Impact of Event Scale-Revised; PHQ-9, Patient Health Questionnaire-9; SD, standard deviation.

1 PHQ-9 (0 to 27), GAD-7 (0 to 21), and IES-R (0 to 88) are self-administered scales that measure the severity of depression, generalized anxiety disorder, and post-traumatic stress disorder symptoms, respectively; Relative risks were estimated from negative binomial generalized linear mixed models with covariates including age, sex, region, marital status, education level, and average monthly household income; In the models, we used individual identification and the difference in survey periods (in years) from the occurrence of the disaster as random intercept effects to reflect repeated measurements of scores in the case group, up to a maximum of 4 times; β and standard error were based on log-transformed scores of PHQ-9, GAD-7, and IES-R.

2 Changes in household economic status after a disaster in the baseline questionnaire.

## Supplementary materials

Supplementary Material 1. Map of South Korea by major regions.

Supplementary Material 2. Distribution of mental health indicators with elapsed time following a disaster in study participants

Supplementary Material 3. Association between disaster severity variables and the clinical group of depression, anxiety, and post-traumatic stress disorder

Supplementary Material 4. Stratification analyses by potential effect modifiers of the association between disaster severity variables and mental health scores

Supplementary Material 5. Association between disaster severity variables and mental health scores based on responses within 2 years of natural disaster occurrence

Supplementary Material 6. Association between disaster intensity variables and mental health scores excluding the 2018 Pohang earthquake area control

Supplementary Material 7. Association between disaster severity variables and mental health scores: analysis of typhoon and heavy rainfall victim subgroups
