# Supplementary material for: Identifying factors associated with mental health status following climate-related disasters: a nationwide longitudinal panel study in Korea
Source: Epidemiol Health. 2025 Mar 27;47:e2025014. doi: 10.4178/epih.e2025014 (PMC12178763; doi:10.4178/epih.e2025014)
Supplement: Supplementary Material 1. — Map of South Korea by major regions. [file epih-47-e2025014-Supplementary-1.docx]

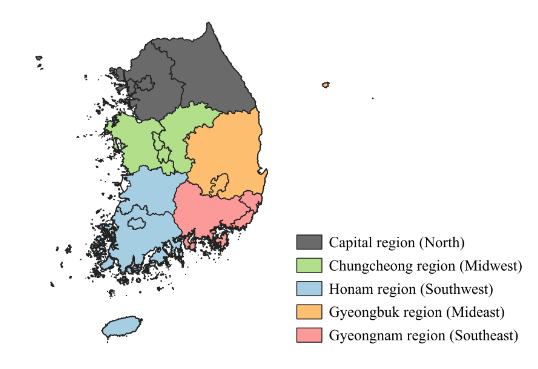


**Supplementary Material 1.** Map of South Korea by major regions. Capital (North), Chungcheong (Midwest), Honam (Southwest), Gyeongbuk (Mideast), and Gyeongnam (Southeast)
