# Supplementary material for: Identifying factors associated with mental health status following climate-related disasters: a nationwide longitudinal panel study in Korea
Source: Epidemiol Health. 2025 Mar 27;47:e2025014. doi: 10.4178/epih.e2025014 (PMC12178763; doi:10.4178/epih.e2025014)
Supplement: Supplementary Material 2. — Distribution of mental health indicators with elapsed time following a disaster in study participants [file epih-47-e2025014-Supplementary-2.docx]

**Supplementary Material 2.** Distribution of mental health indicators with elapsed time following a disaster in study participants

|  | Victim group: elapsed time after disaster occurrence (year) (Observation number = 4925, 100.0%) | | | | | | | | | | | | | | | | | | | | | | | | | | | | | | | Control (N = 893) | | | |  |  |
| --- | --- | --- | --- | --- | --- | --- | --- | --- | --- | --- | --- | --- | --- | --- | --- | --- | --- | --- | --- | --- | --- | --- | --- | --- | --- | --- | --- | --- | --- | --- | --- | --- | --- | --- | --- | --- | --- |
|  | 0 (N = 259, 5.3%) | | | 1 (N = 757, 15.4%) | | | | 2 (N = 1039, 21.1%) | | | | 3 (N = 642, 13.0%) | | | | 4 (N = 965, 19.6%) | | | | 5 (N = 672, 13.6%) | | | | 6 (N = 591, 12.0%) | | | | 7 (N = 591, 12.0%) | | | |  |  |  |  |  |  |
| **Depression** |  |  | |  | |  | |  | |  | |  | |  | |  | |  | |  | |  | |  | |  | |  | |  | |  | |  | | |  |
| PHQ-9 (GM, GSD) | 2.01 | | 1.86 | | 2.17 | | 1.75 | | 2.05 | | 1.67 | | 1.77 | | 1.54 | | 2.94 | | 1.17 | | 1.30 | | 1.57 | | 1.45 | | 1.71 | | 1.38 | | 1.43 | | 1.07 | | 1.24 | | |
| High-risk group (N, %) | 45 | | 17.4 | | 140 | | 18.5 | | 181 | | 17.4 | | 78 | | 12.1 | | 132 | | 13.7 | | 69 | | 10.3 | | 43 | | 13.9 | | 22 | | 7.8 | | 39 | | 4.4 | | |
| **Anxiety** |  | |  | |  | |  | |  | |  | |  | |  | |  | |  | |  | |  | |  | |  | |  | |  | |  | |  | | |
| GAD-7 (GM, GSD) | 1.27 | | 1.54 | | 1.26 | | 1.44 | | 1.25 | | 1.46 | | 1.04 | | 1.34 | | 0.85 | | 1.35 | | 0.77 | | 1.30 | | 0.69 | | 1.32 | | 0.66 | | 1.22 | | 0.71 | | 1.00 | | |
| Clinical group (N, %) | 56 | | 21.6 | | 166 | | 21.9 | | 232 | | 22.3 | | 109 | | 17.0 | | 160 | | 16.6 | | 93 | | 13.8 | | 45 | | 14.5 | | 37 | | 13.2 | | 73 | | 8.2 | | |
| **PTSD** |  | |  | |  | |  | |  | |  | |  | |  | |  | |  | |  | |  | |  | |  | |  | |  | |  | |  | | |
| IES-R (GM, GSD) | 10.28 | | 3.06 | | 7.43 | | 3.28 | | 7.10 | | 3.19 | | 5.47 | | 2.98 | | 3.92 | | 3.12 | | 3.21 | | 2.84 | | 2.51 | | 2.85 | | 4.76 | | 2.67 | | - | | - | | |
| Clinical group (N, %) | 101 | | 39.0 | | 244 | | 32.2 | | 293 | | 28.2 | | 147 | | 22.9 | | 155 | | 16.1 | | 85 | | 12.6 | | 46 | | 14.8 | | 38 | | 13.5 | |  | | - | | |
| GM: geometric mean; GSD: geometric standard deviation; PTSD: Post-traumatic stress disorder High-risk group of depression: PHQ-9 (Patient Health Questionnaire-9) score >= 9; Anxiety clinical group: GAD-7 (Generalized Anxiety Disorder-7) score >= 5; PTSD clinical group: IES-R score (Impact of Event Scale-Revised) >= 24. | | | | | | | | | | | | | | | | | | | | | | | | | | | | | | | | | | | |  |  |
