# Supplementary material for: Identifying factors associated with mental health status following climate-related disasters: a nationwide longitudinal panel study in Korea
Source: Epidemiol Health. 2025 Mar 27;47:e2025014. doi: 10.4178/epih.e2025014 (PMC12178763; doi:10.4178/epih.e2025014)
Supplement: Supplementary Material 3. — Association between disaster severity variables and the clinical group of depression, anxiety, and post-traumatic stress disorder [file epih-47-e2025014-Supplementary-3.docx]

**Supplementary Material 3.** Association between disaster severity variables and the clinical group of depression, anxiety, and post-traumatic stress disorder

| Total surveyed N = 5,818 | High-risk group of depression (N = 749, 12.9%) | | Anxiety clinical group (N = 971, 16.7%) | | PTSD clinical group (N = 1,109, 19.1%) | |
| --- | --- | --- | --- | --- | --- | --- |
|  | N (%) | OR (95%CIs) | N (%) | OR (95%CIs) | N (%) | OR (95%CIs) |
| **Control** | 39 (4.4) | Ref. | 73 (8.2) | Ref. | - | - |
| **Casualties experienced by oneself or nearby** | | | | | | |
| No | 478 (11.8) | 2.92 (1.79, 4.75) | 620 (15.4) | 1.65 (1.15, 2.37) | 811 (19.5) | Ref. |
| Yes | 232 (26.1) | 14.88 (8.08, 27.38) | 278 (31.3) | 6.33 (3.99, 10.03) | 384 (41.4) | 3.49 (2.71, 4.51) |
| **Self-reported disaster-induced losses** | | | | | | |
| ≤Moderate | 187 (12.1) | 2.45 (1.45, 4.15) | 240 (15.5) | 1.81 (1.20, 2.73) | 294 (18.5) | Ref. |
| High | 223 (12.8) | 2.48 (1.47, 4.18) | 290 (16.6) | 1.82 (1.21, 2.73) | 427 (23.6) | 1.26 (0.98, 1.63) |
| Very high | 231 (17.2) | 4.49 (2.59, 7.81) | 288 (21.5) | 3.04 (1.97, 4.68) | 373 (26.7) | 1.67 (1.27, 2.19) |
| **Relocation, separation from family, and residing in temporary housing** | | | | | | |
| No | 552 (13.3) | 2.43 (1.50, 3.96) | 716 (17.2) | 2.21 (1.52, 3.22) | 886 (20.7) | Ref. |
| Yes | 158 (20.8) | 6.17 (3.38, 11.25) | 182 (24.0) | 4.96 (3.06, 8.05) | 309 (38.1) | 2.32 (1.78, 3.02) |
| **Household income^1^** | | | | | | |
| No change / Increased | 391 (11.1) | 1.96 (1.21, 3.20) | 525 (14.9) | 1.65 (1.14, 2.38) | 712 (20.1) | Ref. |
| Decreased | 319 (22.9) | 6.77 (4.02, 11.39) | 373 (26.8) | 4.67 (3.11, 7.01) | 397 (28.6) | 2.19 (1.78, 2.70) |
| **Household asset^1^** | | | | | | |
| No change / Increased | 479 (11.8) | 2.63 (1.66, 4.16) | 615 (15.2) | 1.73 (1.20, 2.50) | 795 (19.7) | Ref. |
| Decreased | 231 (26.2) | 7.98 (4.78, 13.32) | 283 (32.1) | 5.81 (3.80, 8.89) | 314 (35.6) | 2.57 (2.06, 3.20) |
| **Household debt^1^** | | | | | | |
| No change / Decreased | 577 (13.7) | 2.69 (1.66, 4.36) | 713 (16.9) | 1.92 (1.33, 2.78) | 866 (20.6) | Ref. |
| Increased | 133 (18.7) | 5.45 (3.15, 9.43) | 185 (26.0) | 4.92 (3.19, 7.57) | 243 (34.1) | 2.33 (1.83, 2.96) |
| High-risk depression: PHQ-9 (Patient Health Questionnaire-9) score >= 9; Anxiety clinical group: GAD-7 (Generalized Anxiety Disorder-7) score >= 5; PTSD clinical group: IES-R score (Impact of Event Scale-Revised) >= 24.  The ORs were estimated from binomial generalized linear mixed models with covariates including age, gender, region, marital status, education, and average monthly household income. In the models, we used individual identification and the difference in survey periods (in years) from the occurrence of the disaster as random intercept effects to reflect repeated measurements of scores in the case group, up to a maximum of 4 times.  ^1^ These indicate the changes in household economic status after a disaster in the baseline questionnaire. | | | | | | |
