# Supplementary material for: Identifying factors associated with mental health status following climate-related disasters: a nationwide longitudinal panel study in Korea
Source: Epidemiol Health. 2025 Mar 27;47:e2025014. doi: 10.4178/epih.e2025014 (PMC12178763; doi:10.4178/epih.e2025014)
Supplement: Supplementary Material 4. — Stratification analyses by potential effect modifiers of the association between disaster severity variables and mental health scores [file epih-47-e2025014-Supplementary-4.docx]

**Supplementary Material 4.** Stratification analyses by potential effect modifiers of the association between disaster severity variables and mental health scores

| **Strata** | | **PHQ-9** | | | | **GAD-7** | | | | **IES-R** | | | |
| --- | --- | --- | --- | --- | --- | --- | --- | --- | --- | --- | --- | --- | --- |
|  |  | Beta (SE) | p-value | Beta (SE) | p-value | Beta (SE) | p-value | Beta (SE) | p-value | Beta (SE) | p-value | Beta (SE) | p-value |
| **Age** | | **(<65 years)** | | **(>=65 years)** | | **(<65 years)** | | **(>=65 years)** | | **(<65 years)** | | **(>=65 years)** | |
|  | Controls | Ref. | | Ref. | | Ref. | | Ref. | | - | | - | |
|  | Casualties including oneself and those nearby | | | | | | | | | | | |  |
|  | No | 0.31 (0.14) | 0.023 | 0.45 (0.16) | 0.004 | 0.10 (0.16) | 0.503 | 0.57 (0.23) | 0.013 | Ref. | | Ref. | |
|  | Yes | 0.79 (0.15) | <0.001 | 0.93 (0.17) | <0.001 | 0.78 (0.18) | <0.001 | 1.39 (0.26) | <0.001 | 0.51 (0.09) | <0.001 | 0.59 (0.09) | <0.001 |
|  | Self-reported disaster-induced losses | | | | | | | | | | | |  |
|  | Moderate or less | 0.31 (0.15) | 0.032 | 0.49 (0.16) | 0.003 | 0.15 (0.16) | 0.356 | 0.66 (0.24) | 0.007 | Ref. | | Ref. | |
|  | Significant | 0.36 (0.15) | 0.013 | 0.53 (0.16) | 0.001 | 0.14 (0.16) | 0.404 | 0.63 (0.24) | 0.009 | 0.10 (0.08) | 0.199 | 0.00 (0.08) | 0.975 |
|  | Very significant | 0.51 (0.15) | <0.001 | 0.64 (0.17) | <0.001 | 0.38 (0.16) | 0.020 | 0.91 (0.25) | <0.001 | 0.29 (0.09) | 0.001 | 0.20 (0.10) | 0.038 |
|  | Relocation, separation from family, and residing in temporary housing | | | | | | | | | | | |  |
|  | No | 0.28 (0.14) | 0.042 | 0.52 (0.16) | 0.001 | 0.11 (0.15) | 0.460 | 0.65 (0.20) | 0.001 | Ref. | | Ref. | |
|  | Yes | 0.76 (0.15) | <0.001 | 0.56 (0.18) | 0.002 | 0.61 (0.17) | <0.001 | 0.91 (0.25) | <0.001 | 0.39 (0.08) | <0.001 | 0.31 (0.12) | 0.009 |
|  | Household income^1^ | | | | | | | | | | | |  |
|  | No change / Increased | 0.27 (0.14) | 0.053 | 0.40 (0.16) | 0.010 | 0.06 (0.15) | 0.687 | 0.54 (NA) | NA | Ref. | | Ref. | |
|  | Decreased | 0.88 (0.15) | <0.001 | 0.85 (0.16) | <0.001 | 0.77 (0.17) | <0.001 | 1.05 (NA) | NA | 0.45 (0.08) | <0.001 | 0.34 (0.07) | <0.001 |
|  | Household asset^1^ | | | | | | | | | | | |  |
|  | No change / Increased | 0.31 (0.14) | 0.027 | 0.43 (0.15) | 0.006 | 0.10 (0.16) | 0.510 | 0.53 (0.23) | 0.020 | Ref. | | Ref. | |
|  | Decreased | 1.00 (0.15) | <0.001 | 0.88 (0.16) | <0.001 | 0.98 (0.18) | <0.001 | 1.12 (0.24) | <0.001 | 0.54 (0.09) | <0.001 | 0.42 (0.08) | <0.001 |
|  | Household debt^1^ | | | | | | | | | | | |  |
|  | No change / Decreased | 0.35 (NA) | NA | 0.52 (0.16) | 0.001 | 0.11 (0.15) | 0.476 | 0.68 (0.24) | 0.004 | Ref. | | Ref. | |
|  | Increased | 0.75 (NA) | NA | 0.89 (0.19) | <0.001 | 0.70 (0.17) | <0.001 | 1.13 (0.28) | <0.001 | 0.35 (0.08) | <0.001 | 0.38 (0.13) | 0.003 |
| **Sex** | | **(Male)** | | **(Female)** | | **(Male)** | | **(Female)** | | **(Male)** | | **(Female)** | |
|  | Controls | Ref. | | Ref. | | Ref. | | Ref. | | - | | - | |
|  | Casualties including oneself and those nearby | | | | | | | | | | | |  |
|  | No | 0.43 (0.15) | 0.005 | 0.34 (0.14) | 0.013 | 0.14 (0.18) | 0.435 | 0.34 (0.18) | 0.052 | Ref. | | Ref. | |
|  | Yes | 0.79 (0.17) | <0.001 | 0.94 (0.16) | <0.001 | 0.68 (0.21) | 0.001 | 1.20 (0.20) | <0.001 | 0.44 (0.09) | <0.001 | 0.58 (0.09) | <0.001 |
|  | Self-reported disaster-induced losses | | | | | | | | | | | |  |
|  | Moderate or less | 0.46 (0.16) | 0.004 | 0.37 (0.15) | 0.012 | 0.24 (0.20) | 0.219 | 0.36 (0.18) | 0.047 | Ref. | | Ref. | |
|  | Significant | 0.47 (0.16) | 0.003 | 0.44 (0.15) | 0.002 | 0.13 (0.19) | 0.500 | 0.42 (0.18) | 0.020 | 0.03 (0.08) | 0.764 | 0.05 (0.08) | 0.555 |
|  | Very significant | 0.62 (0.16) | <0.001 | 0.55 (0.15) | <0.001 | 0.39 (0.20) | 0.051 | 0.65 (0.19) | <0.001 | 0.27 (0.09) | 0.003 | 0.18 (0.09) | 0.048 |
|  | Relocation, separation from family, and residing in temporary housing | | | | | | | | | | | |  |
|  | No | 0.43 (0.15) | 0.005 | 0.35 (0.14) | 0.011 | 0.16 (0.18) | 0.374 | 0.36 (NA) | NA | Ref. | | Ref. | |
|  | Yes | 0.72 (0.17) | <0.001 | 0.76 (0.16) | <0.001 | 0.53 (0.20) | 0.010 | 0.87 (NA) | NA | 0.24 (0.10) | 0.014 | 0.43 (0.09) | <0.001 |
|  | Household income^1^ | | | | | | | | | | | |  |
|  | No change / Increased | 0.40 (0.15) | 0.008 | 0.32 (0.14) | 0.019 | 0.13 (0.19) | 0.502 | 0.26 (0.17) | 0.118 | Ref. | | Ref. | |
|  | Decreased | 0.99 (0.16) | <0.001 | 0.79 (0.14) | <0.001 | 0.67 (0.20) | 0.001 | 0.94 (0.17) | <0.001 | 0.38 (0.08) | <0.001 | 0.36 (0.07) | <0.001 |
|  | Household asset^1^ | | | | | | | | | | | |  |
|  | No change / Increased | 0.39 (0.15) | 0.010 | 0.35 (0.14) | 0.011 | 0.08 (0.18) | 0.658 | 0.37 (0.18) | 0.037 | Ref. | | Ref. | |
|  | Decreased | 1.00 (0.17) | <0.001 | 0.90 (0.15) | <0.001 | 0.84 (0.19) | <0.001 | 1.11 (0.19) | <0.001 | 0.50 (0.09) | <0.001 | 0.46 (0.08) | <0.001 |
|  | Household debt^1^ | | | | | | | | | | | |  |
|  | No change / Decreased | 0.43 (0.15) | 0.004 | 0.43 (0.14) | 0.002 | 0.06 (0.18) | 0.738 | 0.44 (0.18) | 0.015 | Ref. | | Ref. | |
|  | Increased | 0.88 (0.17) | <0.001 | 0.67 (0.16) | <0.001 | 0.74 (0.19) | <0.001 | 0.87 (0.21) | <0.001 | 0.44 (0.09) | <0.001 | 0.25 (0.09) | 0.007 |
| **Marital status** | | **(Single)** | | **(M + S/D/W)** | | **(Single)** | | **(M + S/D/W)** | | **(Single)** | | **(M + S/D/W)** | |
|  | Controls | Ref. | | Ref. | | Ref. | | Ref. | | - | | - | |
|  | Casualties including oneself and those nearby | | | | | | | | | | | |  |
|  | No | 0.12 (NA) | NA | 0.42 (0.11) | <0.001 | -0.28 (NA) | NA | 0.42 (NA) | NA | Ref. | | Ref. | |
|  | Yes | 0.52 (NA) | NA | 0.90 (0.12) | <0.001 | 0.44 (NA) | NA | 1.12 (NA) | NA | 0.33 (0.18) | 0.078 | 0.56 (0.07) | <0.001 |
|  | Self-reported disaster-induced losses | | | | | | | | | | | |  |
|  | Moderate or less | -0.08 (0.29) | 0.781 | 0.48 (0.12) | <0.001 | -0.28 (NA) | NA | 0.52 (0.16) | 0.001 | Ref. | | Ref. | |
|  | Significant | 0.13 (0.29) | 0.658 | 0.51 (0.12) | <0.001 | -0.20 (NA) | NA | 0.50 (0.16) | 0.002 | 0.16 (0.16) | 0.312 | 0.06 (0.06) | 0.374 |
|  | Very significant | 0.38 (0.29) | 0.187 | 0.60 (0.12) | <0.001 | 0.09 (NA) | NA | 0.70 (0.16) | <0.001 | 0.45 (0.18) | 0.013 | 0.22 (0.07) | 0.001 |
|  | Relocation, separation from family, and residing in temporary housing | | | | | | | | | | | |  |
|  | No | 0.09 (0.28) | 0.747 | 0.44 (NA) | NA | -0.21 (0.30) | 0.490 | 0.44 (0.16) | 0.005 | Ref. | | Ref. | |
|  | Yes | 0.51 (0.31) | 0.097 | 0.77 (NA) | NA | 0.09 (0.35) | 0.799 | 0.89 (0.17) | <0.001 | 0.37 (0.17) | 0.027 | 0.34 (0.07) | <0.001 |
|  | Household income^1^ | | | | | | | | | | | |  |
|  | No change / Increased | 0.16 (NA) | NA | 0.36 (NA) | NA | -0.20 (0.29) | 0.501 | 0.41 (0.15) | 0.008 | Ref. | | Ref. | |
|  | Decreased | 0.54 (NA) | NA | 0.90 (NA) | NA | 0.13 (0.35) | 0.714 | 1.05 (0.16) | <0.001 | 0.37 (0.19) | 0.049 | 0.36 (0.06) | <0.001 |
|  | Household asset^1^ | | | | | | | | | | | |  |
|  | No change / Increased | 0.16 (0.28) | 0.572 | 0.41 (0.11) | <0.001 | -0.22 (0.29) | 0.444 | 0.45 (0.15) | 0.003 | Ref. | | Ref. | |
|  | Decreased | 0.59 (0.34) | 0.077 | 0.99 (0.12) | <0.001 | 0.41 (0.35) | 0.242 | 1.21 (0.16) | <0.001 | 0.46 (0.20) | 0.018 | 0.46 (0.06) | <0.001 |
|  | Household debt^1^ | | | | | | | | | | | |  |
|  | No change / Decreased | 0.17 (0.28) | 0.534 | 0.47 (0.11) | <0.001 | -0.21 (0.29) | 0.462 | 0.49 (0.16) | 0.002 | Ref. | | Ref. | |
|  | Increased | 0.28 (0.32) | 0.382 | 0.88 (0.12) | <0.001 | 0.24 (0.34) | 0.482 | 1.04 (0.17) | <0.001 | 0.17 (0.17) | 0.334 | 0.40 (0.07) | <0.001 |
| **Education** | | **(>= High school)** | | **(<= Middle school)** | | **(>= High school)** | | **(<= Middle school)** | | **(>= High school)** | | **(<= Middle school)** | |
|  | Controls | Ref. | | Ref. | | Ref. | | Ref. | | - | | - | |
|  | Casualties including oneself and those nearby | | | | | | | | | | | |  |
|  | No | 0.28 (0.15) | 0.056 | 0.49 (0.15) | 0.001 | 0.13 (NA) | NA | 0.37 (0.21) | 0.084 | Ref. | | Ref. | |
|  | Yes | 0.74 (0.16) | <0.001 | 0.98 (0.17) | <0.001 | 0.78 (NA) | NA | 1.18 (0.25) | <0.001 | 0.52 (0.08) | <0.001 | 0.55 (0.09) | <0.001 |
|  | Self-reported disaster-induced losses | | | | | | | | | | | |  |
|  | Moderate or less | 0.36 (0.16) | 0.022 | 0.46 (0.15) | 0.003 | 0.21 (0.17) | 0.212 | 0.42 (NA) | NA | Ref. | | Ref. | |
|  | Significant | 0.30 (0.15) | 0.048 | 0.59 (0.15) | <0.001 | 0.12 (0.17) | 0.487 | 0.45 (NA) | NA | 0.07 (0.08) | 0.364 | 0.05 (0.08) | 0.568 |
|  | Very significant | 0.54 (0.16) | 0.001 | 0.63 (0.16) | <0.001 | 0.52 (0.17) | 0.003 | 0.54 (NA) | NA | 0.33 (0.09) | <0.001 | 0.20 (0.09) | 0.024 |
|  | Relocation, separation from family, and residing in temporary housing | | | | | | | | | | | |  |
|  | No | 0.27 (0.15) | 0.068 | 0.52 (0.15) | <0.001 | 0.16 (0.16) | 0.306 | 0.39 (NA) | NA | Ref. | | Ref. | |
|  | Yes | 0.71 (0.16) | <0.001 | 0.69 (0.17) | <0.001 | 0.60 (0.18) | 0.001 | 0.77 (NA) | NA | 0.38 (0.08) | <0.001 | 0.27 (0.11) | 0.015 |
|  | Household income^1^ | | | | | | | | | | | |  |
|  | No change / Increased | 0.28 (0.15) | 0.051 | 0.40 (0.14) | 0.006 | 0.12 (0.16) | 0.457 | 0.26 (NA) | NA | Ref. | | Ref. | |
|  | Decreased | 0.86 (0.16) | <0.001 | 0.88 (0.15) | <0.001 | 0.80 (0.18) | <0.001 | 0.83 (NA) | NA | 0.47 (0.08) | <0.001 | 0.32 (0.07) | <0.001 |
|  | Household asset^1^ | | | | | | | | | | | |  |
|  | No change / Increased | 0.32 (0.15) | 0.027 | 0.41 (0.14) | 0.005 | 0.16 (0.16) | 0.318 | 0.22 (0.16) | 0.171 | Ref. | | Ref. | |
|  | Decreased | 0.93 (0.16) | <0.001 | 0.96 (0.15) | <0.001 | 0.97 (0.19) | <0.001 | 0.92 (0.17) | <0.001 | 0.54 (0.09) | <0.001 | 0.45 (0.08) | <0.001 |
|  | Household debt^1^ | | | | | | | | | | | |  |
|  | No change / Decreased | 0.34 (0.15) | 0.021 | 0.53 (0.15) | <0.001 | 0.16 (0.16) | 0.322 | 0.43 (0.22) | 0.047 | Ref. | | Ref. | |
|  | Increased | 0.75 (0.16) | <0.001 | 0.86 (0.17) | <0.001 | 0.75 (0.18) | <0.001 | 0.91 (0.25) | <0.001 | 0.40 (0.08) | <0.001 | 0.31 (0.11) | 0.005 |
| **Average monthly household income** | | **(>= 2M KRW)** | | **(< 2M KRW)** | | **(>= 2M KRW)** | | **(< 2M KRW)** | | **(>= 2M KRW)** | | **(< 2M KRW)** | |
|  | Controls | Ref. | | Ref. | | Ref. | | Ref. | | - | | - | |
|  | Casualties including oneself and those nearby | | | | | | | | | | | |  |
|  | No | 0.35 (0.15) | 0.016 | 0.42 (0.16) | 0.008 | 0.12 (NA) | NA | 0.60 (0.23) | 0.009 | Ref. | | Ref. | |
|  | Yes | 0.74 (0.16) | <0.001 | 0.99 (0.17) | <0.001 | 0.73 (NA) | NA | 1.45 (0.26) | <0.001 | 0.42 (0.08) | <0.001 | 0.62 (0.09) | <0.001 |
|  | Self-reported disaster-induced losses | | | | | | | | | | | |  |
|  | Moderate or less | 0.47 (0.15) | 0.002 | 0.35 (0.17) | 0.035 | 0.30 (0.18) | 0.104 | 0.55 (NA) | NA | Ref. | | Ref. | |
|  | Significant | 0.35 (0.15) | 0.022 | 0.55 (0.16) | 0.001 | 0.08 (0.18) | 0.664 | 0.76 (NA) | NA | -0.05 (0.08) | 0.513 | 0.19 (0.09) | 0.031 |
|  | Very significant | 0.46 (0.15) | 0.003 | 0.73 (0.17) | <0.001 | 0.33 (0.19) | 0.076 | 1.01 (NA) | NA | 0.10 (0.08) | 0.238 | 0.46 (0.10) | <0.001 |
|  | Relocation, separation from family, and residing in temporary housing | | | | | | | | | | | |  |
|  | No | 0.31 (0.15) | 0.037 | 0.47 (NA) | NA | 0.11 (NA) | NA | 0.68 (0.23) | 0.003 | Ref. | | Ref. | |
|  | Yes | 0.71 (0.16) | <0.001 | 0.75 (NA) | NA | 0.60 (NA) | NA | 1.02 (0.26) | <0.001 | 0.31 (0.08) | <0.001 | 0.36 (0.11) | 0.001 |
|  | Household income^1^ | | | | | | | | | | | |  |
|  | No change / Increased | 0.31 (0.14) | 0.030 | 0.36 (0.16) | 0.021 | 0.09 (0.17) | 0.577 | 0.53 (0.23) | 0.023 | Ref. | | Ref. | |
|  | Decreased | 0.84 (0.15) | <0.001 | 0.89 (0.16) | <0.001 | 0.66 (0.18) | <0.001 | 1.20 (0.24) | <0.001 | 0.38 (0.07) | <0.001 | 0.39 (0.08) | <0.001 |
|  | Household asset^1^ | | | | | | | | | | | |  |
|  | No change / Increased | 0.35 (0.14) | 0.016 | 0.39 (0.16) | 0.012 | 0.13 (NA) | NA | 0.49 (0.23) | 0.034 | Ref. | | Ref. | |
|  | Decreased | 0.91 (0.16) | <0.001 | 0.96 (0.16) | <0.001 | 0.84 (NA) | NA | 1.27 (0.24) | <0.001 | 0.47 (0.09) | <0.001 | 0.47 (0.09) | <0.001 |
|  | Household debt^1^ | | | | | | | | | | | |  |
|  | No change / Decreased | 0.39 (0.14) | 0.008 | 0.53 (0.16) | 0.001 | 0.16 (0.17) | 0.350 | 0.74 (0.23) | 0.002 | Ref. | | Ref. | |
|  | Increased | 0.66 (0.16) | <0.001 | 1.12 (0.19) | <0.001 | 0.62 (0.19) | 0.001 | 1.52 (0.27) | <0.001 | 0.35 (0.08) | <0.001 | 0.40 (0.12) | 0.001 |
| GAD-7: Generalized Anxiety Disorder-7; IES-R: Impact of Event Scale-Revised; KRW: Korean Won; M + D/S/W: Married + separated/divorced/widowed; NA: not applicable: standard error was not estimated in the model; PHQ-9: Patient Health Questionnaire-9; S: single; SE: standard error  PHQ-9 (0 to 27), GAD-7 (0 to 21), and IES-R (0 to 88) are self-administered scales that measure the severity of depression, generalized anxiety disorder, and post-traumatic stress disorder symptoms, respectively. The betas and standard errors were estimated from negative binomial generalized linear mixed models with covariates including age, gender, region, marital status, education, and average monthly household income. In the models, we used individual identification and the difference in survey periods (in years) from the occurrence of the disaster as random intercept effects to reflect repeated measurements of scores in the case group, up to a maximum of 4 times.  ^1^ These indicate the changes in household economic status after a disaster in the baseline questionnaire. | | | | | | | | | | | | | |
