# Supplementary material for: Identifying factors associated with mental health status following climate-related disasters: a nationwide longitudinal panel study in Korea
Source: Epidemiol Health. 2025 Mar 27;47:e2025014. doi: 10.4178/epih.e2025014 (PMC12178763; doi:10.4178/epih.e2025014)
Supplement: Supplementary Material 5. — Association between disaster severity variables and mental health scores based on responses within 2 years of natural disaster occurrence [file epih-47-e2025014-Supplementary-5.docx]

**Supplementary Material 5.** Association between disaster severity variables and mental health scores based on responses within 2 years of natural disaster occurrence

| Disaster intensity variables | Total N =  2,130 (100.0%) | Surveyed N = 2,948 (100.0%) | PHQ-9 (Mean ± SD: 1.7 ± 1.6) | | | | GAD-7 (Mean ± SD: 1.1 ± 1.3) | | | | IES-R (Mean ± SD: 3.5 ± 3.7) | | | |
| --- | --- | --- | --- | --- | --- | --- | --- | --- | --- | --- | --- | --- | --- | --- |
|  | N (%) | | Mean ± SD | Beta | SE | p-value | Mean ± SD | Beta | SE | p-value | Mean ± SD | Beta | SE | p-value |
| Control | 893 (41.9) | 893 (30.3) | 1.1 ± 1.2 | Ref. |  |  | 0.7 ± 1.0 | Ref. |  |  | - |  |  |  |
| Casualties including oneself and those nearby | | | | | | | | | | | | |  |  |
| No | 977 (45.9) | 1659 (56.3) | 1.9 ± 1.6 | 0.47 | 0.07 | <0.001 | 1.1 ± 1.4 | 0.44 | 0.09 | <0.001 | 6.4 ± 3.1 | Ref. |  |  |
| Yes | 260 (12.2) | 396 (13.4) | 3.3 ± 1.9 | 0.99 | 0.10 | <0.001 | 2.1 ± 1.7 | 1.12 | 0.12 | <0.001 | 14.9 ± 2.9 | 0.45 | 0.08 | <0.001 |
| Self-reported disaster-induced losses | | | | | | | | | | | | |  |  |
| ≤Moderate | 376 (18.4) | 683 (23.8) | 1.7 ± 1.7 | 0.48 | 0.09 | <0.001 | 1.0 ± 1.3 | 0.39 | 0.11 | <0.001 | 5.6 ± 3.1 | Ref. |  |  |
| High | 465 (22.7) | 751 (26.2) | 2.0 ± 1.7 | 0.55 | 0.09 | <0.001 | 1.3 ± 1.5 | 0.57 | 0.10 | <0.001 | 7.6 ± 3.2 | 0.13 | 0.08 | 0.087 |
| Very high | 314 (15.3) | 539 (18.8) | 2.4 ± 1.7 | 0.73 | 0.10 | <0.001 | 1.5 ± 1.5 | 0.82 | 0.11 | <0.001 | 10.1 ± 3.1 | 0.33 | 0.09 | <0.001 |
| Relocation, separation from family, and residing in temporary housing | | | | | | | | | | | | |  |  |
| No | 972 (45.6) | 1590 (53.9) | 2.0 ± 1.7 | 0.50 | 0.07 | <0.001 | 1.2 ± 1.4 | 0.48 | 0.09 | <0.001 | 6.8 ± 3.2 | Ref. |  |  |
| Yes | 265 (12.4) | 465 (15.8) | 2.3 ± 1.9 | 0.82 | 0.10 | <0.001 | 1.5 ± 1.6 | 0.86 | 0.12 | <0.001 | 10.6 ± 3.1 | 0.30 | 0.08 | <0.001 |
| Household income^1^ | | | | | | | | | | | | |  |  |
| No change / Increased | 911 (42.8) | 1542 (52.3) | 1.8 ± 1.7 | 0.43 | 0.07 | <0.001 | 1.1 ± 1.4 | 0.42 | 0.09 | <0.001 | 6.4 ± 3.3 | Ref. |  |  |
| Decreased | 326 (15.3) | 513 (17.4) | 3.4 ± 1.6 | 0.99 | 0.09 | <0.001 | 2.0 ± 1.6 | 0.99 | 0.10 | <0.001 | 12.1 ± 2.8 | 0.38 | 0.07 | <0.001 |
| Household asset^1^ | | | | | | | | | | | | |  |  |
| No change / Increased | 973 (45.7) | 1648 (55.9) | 1.9 ± 1.7 | 0.49 | 0.07 | <0.001 | 1.1 ± 1.4 | 0.46 | 0.09 | <0.001 | 6.6 ± 3.3 | Ref. |  |  |
| Decreased | 264 (12.4) | 407 (13.8) | 3.2 ± 1.7 | 0.93 | 0.09 | <0.001 | 2.0 ± 1.5 | 0.97 | 0.10 | <0.001 | 13.1 ± 2.6 | 0.35 | 0.08 | <0.001 |
| Household debt^1^ | | | | | | | | | | | | |  |  |
| No change / Decreased | 1009 (47.4) | 1678 (56.9) | 2.1 ± 1.7 | 0.52 | 0.07 | <0.001 | 1.2 ± 1.5 | 0.49 | 0.09 | <0.001 | 7.3 ± 3.2 | Ref. |  |  |
| Increased | 228 (10.7) | 377 (12.8) | 2.1 ± 1.7 | 0.75 | 0.10 | <0.001 | 1.5 ± 1.5 | 0.86 | 0.11 | <0.001 | 9.1 ± 3.2 | 0.28 | 0.08 | 0.001 |
| GAD-7: Generalized Anxiety Disorder-7; IES-R: Impact of Event Scale-Revised; PHQ-9: Patient Health Questionnaire-9; SD: standard deviation; SE: standard error  PHQ-9 (0 to 27), GAD-7 (0 to 21), and IES-R (0 to 88) are self-administered scales that measure the severity of depression, generalized anxiety disorder, and post-traumatic stress disorder symptoms, respectively. The betas and standard errors were estimated from negative binomial generalized linear mixed models with covariates including age, gender, region, marital status, education, and average monthly household income. In the models, we used individual identification and the difference in survey periods (in years) from the occurrence of the disaster as random intercept effects to reflect repeated measurements of scores in the case group, up to a maximum of 4 times.  ^1^ These indicate the changes in household economic status after a disaster in the baseline questionnaire. | | | | | | | | | | | | | | |
