# Supplementary material for: Identifying factors associated with mental health status following climate-related disasters: a nationwide longitudinal panel study in Korea
Source: Epidemiol Health. 2025 Mar 27;47:e2025014. doi: 10.4178/epih.e2025014 (PMC12178763; doi:10.4178/epih.e2025014)
Supplement: Supplementary Material 6. — Association between disaster intensity variables and mental health scores excluding the 2018 Pohang earthquake area control [file epih-47-e2025014-Supplementary-6.docx]

**Supplementary Material 6. Association between disaster intensity variables and mental health scores excluding the 2018 Pohang earthquake area control**

| Disaster intensity variables | PHQ-9 | | | GAD-7 | | |
| --- | --- | --- | --- | --- | --- | --- |
|  | Beta | SE | p-value | Beta | SE | p-value |
| Control | Ref. |  |  | Ref. |  |  |
| Casualties experienced by oneself or nearby | | | | | | |
| No | 0.35 | NA | NA | 0.33 | 0.14 | 0.019 |
| Yes | 0.84 | NA | NA | 1.05 | 0.16 | <0.001 |
| Self-reported disaster-induced losses | | | | | | |
| ≤Moderate | 0.38 | 0.11 | <0.001 | 0.43 | 0.15 | 0.005 |
| High | 0.43 | 0.11 | <0.001 | 0.40 | 0.15 | 0.007 |
| Very high | 0.56 | 0.11 | <0.001 | 0.62 | 0.15 | <0.001 |
| Relocation, separation from family, and residing in temporary housing | | | | | | |
| No | 0.36 | 0.00 | <0.001 | 0.36 | NA | NA |
| Yes | 0.72 | 0.00 | <0.001 | 0.80 | NA | NA |
| Household income^1^ | | | | | | |
| No change / Increased | 0.34 | NA | NA | 0.33 | 0.14 | 0.022 |
| Decreased | 0.87 | NA | NA | 0.95 | 0.15 | <0.001 |
| Household asset^1^ | | | | | | |
| No change / Increased | 0.32 | 0.11 | <0.001 | 0.34 | 0.14 | 0.016 |
| Decreased | 0.91 | 0.12 | <0.001 | 1.10 | 0.15 | <0.001 |
| Household debt^1^ | | | | | | |
| No change / Decreased | 0.40 | 0.11 | <0.001 | 0.39 | 0.14 | 0.007 |
| Increased | 0.77 | 0.12 | <0.001 | 0.93 | 0.16 | <0.001 |
| GAD-7: Generalized Anxiety Disorder-7; PHQ-9: Patient Health Questionnaire-9; Ref.: reference group; RR: relative risk; SD: standard deviation; SE: standard error PHQ-9 (0 to 27) and GAD-7 (0 to 21) are self-administered scales that measure the severity of depression, generalized anxiety disorder, and post-traumatic stress disorder symptoms, respectively. IES-R was not analyzed as it was administered only to disaster victims.  This analysis excludes 188 control participants from the 2018 Pohang earthquake area, which served as the control group in the original study.  The RRs were estimated from negative binomial generalized linear mixed models with covariates including age, sex, region, marital status, education, and average monthly household income. In the models, we used individual identification and the difference in survey periods (in years) from the occurrence of the disaster as random intercept effects to reflect repeated measurements of scores in the case group, up to a maximum of 4 times.  ^1^ These indicate the changes in household economic status after a disaster in the baseline questionnaire. | | | | | | |
|  |  |  |  |  |  |  |
